# Serum Levels of Natriuretic Peptides in Children before and after Treatment for an Atrial Septal Defect, a Patent Ductus Arteriosus, and a Coarctation of the Aorta—A Prospective Study

**DOI:** 10.1155/2010/674575

**Published:** 2010-04-20

**Authors:** Anneli Eerola, Eero Jokinen, Talvikki Boldt, Ilkka P. Mattila, Jaana I. Pihkala

**Affiliations:** ^1^Department of Pediatrics, University Hospital of Tampere, 33521 Tampere, Finland; ^2^Department of Pediatric Cardiology, Hospital for Children and Adolescents, University of Helsinki, Helsinki, Finland; ^3^Department of Pediatric Cardiac Surgery, Hospital for Children and Adolescents, University of Helsinki, Helsinki, Finland

## Abstract

*Background and Objectives*. We evaluated and compared the influence of treatment for atrial septal defect (ASD), patent ductus arteriosus (PDA), and coarctation of the aorta (CoA) on serum levels of N-terminal proatriopeptide and N-terminal probrain natriuretic peptide. Correlations between peptide levels and echocardiographic measurements were calculated. *Patients and Methods*. Peptide levels were measured and echocardiography performed before and 6–12 months after treatment in 21 children with ASD, 25 with PDA, 15 with CoA, and 76 control children. *Results*. ANPN levels were higher than in controls at baseline in all patient groups, and NT-proBNP in patients with ASD and PDA. Both peptide levels were elevated 6 months after treatment and decreased thereafter. Peptide levels were higher in patients with volume than pressure overload. They correlated with echocardiographic measurements. At the 6-month follow-up, dimensions of the originally overloaded ventricle had normalized only in patients with PDA. *Conclusions*. After intervention, peptide levels decrease but normalization takes over 6 months. The type of correlation between peptide levels and echocardiography varies according to the loading condition. Measurement of peptide levels can be used for monitoring the course of a patient's heart disease.

## 1. Introduction

Atrial natriuretic peptide (ANP), brain natriuretic peptide (BNP), C-type natriuretic peptide, and dendroaspis natriuretic peptide form the natriuretic peptide system. They have an important role in regulation of fluid homeostasis, blood pressure/vascular tone, and cardiomyocyte growth. This system functions to retard the progression of heart failure, opposite to the effect of neurohumoral systems like the renin-angiotensin-aldosterone system and the sympathetic nervous system [1, 2].

ANP is produced primarily in the atrial myocyte and in smaller amounts in ventricular myocytes. It appears in fetal ventricular tissue and in hypertrophied ventricles. It is stored as a prohormone in atrial granules and released in response to wall stress. In the presence of heart disease, ANP gene expression is upregulated in ventricular myocytes [2, 3]. BNP is produced in atrial and ventricular myocytes. In healthy people, the main site of gene expression is atrial, but in disease states such as heart failure, ventricular gene expression is upregulated [4, 5]. BNP is stored together with ANP in storage granules in the atrial myocytes in the healthy state. The stimulus for synthesis and release of BNP is wall stress [2, 3].

Normal levels of ANP and BNP and their inactive N-terminal fragment have been investigated using different methods [6–11]. For each method, a specific range of normal values exits. The levels of natriuretic peptides have been shown to be higher immediately after birth and to decrease thereafter, during the first 2–4 months [10–12]. Studies on levels of natriuretic peptides in pediatric patients with different types of congenital heart defects and heart failure have been published. Unfortunately, the follow-up time in most has been short, and study groups have consisted of different types of congenital heart defects [13–16]. To our knowledge, comparison of the effect of treatment for various types of loading conditions on levels of natriuretic peptides has not been performed.

Atrial septal defect (ASD) predisposes the patient to volume overload of the right side of the heart, increased pulmonary vascular resistance, and atrial arrhythmias [17, 18]. The physiological sequelae of ASD depend on the size of the defect, the relation of the diastolic compliances of the right (RV) and left ventricle (LV), and the ratio of pulmonary to systemic vascular resistance [17]. Patent ductus arteriosus (PDA) causes volume overload of the left side of the heart [19] and predisposes the patient to pulmonary hypertension. Coarctation of the aorta (CoA) causes pressure overload of the LV and predisposes the patient to systemic hypertension [20], which may lead to myocardial hypertrophy and diastolic and systolic dysfunction [21].

The timing of treatment for congenital heart defect is based on the hemodynamic and anatomic situation, with consideration of myocardial cell adaptation and chamber remodeling. Therefore, it is important to have multiple methods available for follow-up. The combination of new imaging modalities and measurement of serum levels of natriuretic peptides may allow us to improve evaluation of cardiac function and timing of interventions.

N-terminal proatriopeptide (ANPN) and N-terminal probrain natriuretic peptide (NT-proBNP) may have different regulation of gene expression depending on the form and localization of the stress exerted on the heart. Therefore, treatment for different types of loading condition may cause different types of changes in levels of these peptides. The purpose of this study was to evaluate the influence of treatment for different loading conditions on serum levels of ANPN and NT-proBNP. We correlated peptide levels to two-dimensional (2D) and three -dimensional (3D) echocardiographic findings in these conditions.

## 2. Methods

This prospective follow-up study was carried out at the Hospital for Children and Adolescents, University of Helsinki, Helsinki, Finland, between February 2003 and February 2006. All parents agreed to have their children participate in this clinical trial approved by the hospital ethics committee and gave their written informed consent.

The study groups consisted of 61 patients undergoing percutaneous or surgical treatment for ASD, PDA, or CoA. Twenty-one patients had an ASD, 25 a PDA, and 15 a CoA. The control group comprised 76 healthy voluntary children of whom 64 were 6 months or older. Demographics of children with ASD, PDA, CoA, and controls are shown in Table 1.

All patients and controls underwent clinical cardiovascular examination and blood test sampling for measurement of natriuretic peptides at the time of echocardiographic examinations. All patients were examined just prior to the intervention and 6 months later. The children with ASD and CoA were also examined 12 months after intervention. Control children were examined once. They were asymptomatic and showed no abnormalities in clinical examination, ECG, or echocardiography. 

Serum samples were frozen at −20°C. Serum concentrations of ANPN were measured by immunofluorometric assay. The reagents were manufactured by Medix Biochemica (Espoo, Finland) and the instruments by Delfia Research Fluorometer (Wallac, Turku, Finland). Serum concentrations of NT-proBNP were measured by the electrochemiluminometric method. The reagent kit was manufactured by Roche (Mannheim, Germany), and the samples were analyzed at Limbach Laboratory (Heidelberg, Germany).

### 2.1. Echocardiography

We used the Acuson Sequoia C256 echocardiography system (Siemens, Mountain View, CA) for 2D imaging and for Doppler and M-mode measurements. Three-dimensional echocardiography was performed with TomTec computer software (TomTec Imaging Systems GmHb, Munich, Germany). Two-dimensional echocardiographic examinations were carried out by two observers (A. Eerola and E. Jokinen). All 3D examinations and the analyses of echocardiographic measurements were carried out by one observer (A. Eerola).

### 2.2. 2D Echocardiography

Two-dimensional echocardiography with color flow Doppler was used to evaluate the cardiac anatomy and to measure the size of ASD. M-mode echocardiography was performed from either the parasternal long-axis or short-axis views. RV end-diastolic and LV end-diastolic and end-systolic dimensions were measured, and LV volumes, fractional shortening, and ejection fraction calculated. The *z*-scores of thicknesses of interventricular septum and posterior wall of LV, and those of RV and LV end-diastolic dimensions were determined [22]. The mean of measurements from three cardiac cycles for each participant was saved for analysis.

### 2.3. 3D Echocardiography

Three-dimensional echocardiography was performed with TomTec computer software system, as described earlier in [23]. The time-volume curves obtained served to determine end-diastolic and end-systolic volumes, stroke volume, and ejection fraction.

### 2.4. Cardiac Catheterization

Cardiac catheterization with hemodynamic evaluation and angiography was performed under general endotracheal anesthesia, systemic heparinization, and antimicrobial prophylaxis. In patients with ASD, the procedure was also guided by transesophageal echocardiography. For ASD closure, either the Amplatzer Septal Occluder (AGA Medical Corp., Golden Valley, MN) or the Helex Septal Occluder (W.L. Gore and Associates, Flagstaff, AZ) was used. The PDA was occluded with either Flipper detachable coil (Cook, Bloomington, IN) or with an Amplatzer Duct Occluder (AGA Medical Corp.). In patients with CoA, percutaneous angioplasty was performed using either balloon dilatation only or with stent implantation.

### 2.5. Surgery

Surgical closure of ASD was performed via midline sternotomy with direct suture using normothermic cardiopulmonary bypass. Repair of CoA was performed with resection and end-to-end anastomosis from left thoracotomy.

## 3. Statistical Analysis

Analyses were performed with the Statistical Package for Social Science version 12.01 for Windows (SPSS Inc., Chicago, IL). For variables derived from blood samples, median and range were calculated. Because distribution of parameters tested by Kolmogorov-Smirnov's goodness-of-fit test was not normal, the Mann-Whitney test was used for statistical analysis between groups, and the Wilcoxon Signed-Rank Test for analysis within groups. Spearman's correlation coefficient was used for calculating correlations between the echocardiographic measurements of chamber dimensions and serum concentrations of ANPN and NT-proBNP. The level of significance was *P* < .05.

## 4. Results

### 4.1. Children with ASD

Of the 21 children with ASD, 14 were treated with device closure and 7 with operation. The size of the defect measured a median of 12 (range 7–21) mm in transthoracic echocardiography. No other heart defects were observed. The ASD was closed with the Amplatzer Septal Occluder (AGA Medical Corp., Golden Valley, MN) in 12 patients and with the Helex Septal Occluder (W. L. Gore and Associates, Flagstaff, AZ) in 2 patients. After closure, no residual shunt was evident at the 6- or 12-month follow-up in transthoracic echocardiography.

**Figure 1:**
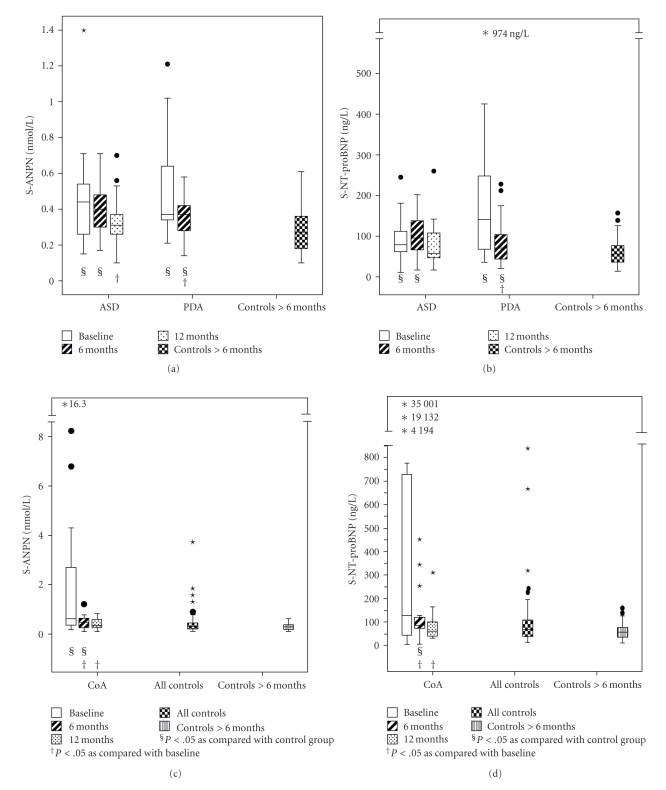
Serum levels of (a) N-terminal proatriopeptide (ANPN) and (b) N-terminal probrain natriuretic peptide (NT-proBNP) in patients with atrial septal defect (ASD) and patent ductus arteriosus (PDA) measured at baseline and at 6 and 12 months' follow-up, and in controls older than six months. Serum levels of (c) N-terminal proatriopeptide (ANPN) and (d) N-terminal probrain natriuretic peptide (NT-proBNP) in patients with coarctation of the aorta measured at baseline and at 6 and 12 months' follow-up, and in all controls and controls older than six months. Whiskers at the top and bottom of each box indicate maximum and minimum; the top and bottom of each box the third and first quartiles; line through the box the median. Solid circles are outliers and ∗indicates an extreme case.

At baseline, children with ASD had higher serum levels of both ANPN and NT-proBNP than controls (Figure 1; Table 2). Six months after occlusion of ASD, the levels were still higher than in controls. Twelve months after intervention, patients no longer differed from controls (Figure 1; Table 2).

**Figure 2:**
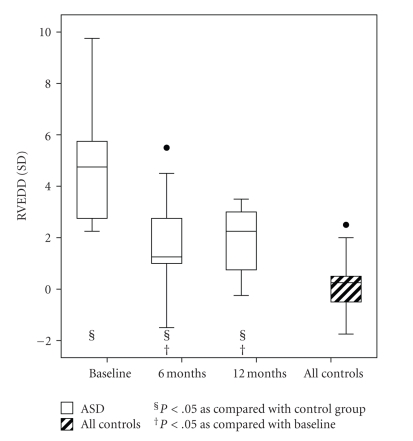
The *z*-score (SD) of end-diastolic diameter of right ventricle (RVEDD) in patients with atrial septal defect (ASD) and in controls. Whiskers at the top and bottom of each box indicate maximum and minimum; the top and bottom of each box the third and first quartiles; line through the box the median. Solid circles are outliers.

During the 12-month follow-up, the end-diastolic diameter of RV decreased after intervention (Table 3). The difference between patients and controls was significant at baseline and remained so up to 12 months after intervention (Figure 2; Table 3). The end-diastolic and systolic diameter and volume of LV increased after cessation of shunting through ASD (Table 3). At baseline, it was smaller than that of controls, with a median of −0.25 (range −1.75–2.00) SD (*P* < .001) but equal to controls at the 6- and 12-month follow-ups. Similarly, the end-diastolic and systolic volumes of LV increased in 3D echocardiography during follow-up (Table 3).

### 4.2. Children with PDA

**Figure 3:**
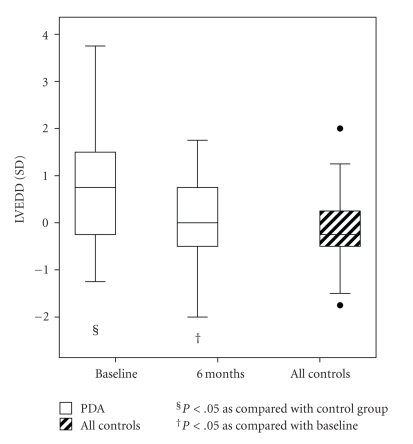
The *z*-score (SD) of end-diastolic diameter of left ventricle (LVEDD) in patients with patent ductus arteriosus (PDA) and in controls. Whiskers at the top and bottom of each box indicate maximum and minimum; the top and bottom of each box the third and first quartiles; line through the box the median. Solid circles are outliers.

All 25 children with PDA underwent percutaneous closure. In 19 children, the PDA was occluded with a Flipper detachable coil (Cook, Bloomington, IN) and in 7 with an Amplatzer Duct Occluder (AGA Medical Corp., Golden Valley, MN). In these children, no other heart defects were observed. One child had a minimal residual shunt 6 months after coil occlusion. Children with PDA had higher levels of ANPN and NT-proBNP than controls before occlusion of PDA. After intervention, levels of both peptides decreased but were still somewhat higher than in controls at the 6-month follow-up (Figure 1, Table 2). The LV end-diastolic and systolic diameter and volume were higher in children with PDA than in controls at baseline, but no difference was seen 6 months after treatment (Figure 3; Table 3). The end-diastolic diameter of RV increased after occlusion of PDA (Table 3).

### 4.3. Children with CoA

Of the 15 children with CoA, 11 had native CoA and 4 recurrent CoA after surgery. Ten patients had a bicuspid aortic valve and one patient had mild mitral valve regurgitation. Five patients underwent balloon dilatation, one of them with a stent implantation. Ten patients were operated on with resection and end-to-end anastomosis. At baseline, the arm-leg blood pressure gradient measured a median of 30 (range 20–56) mm Hg. It decreased to a median of 3 (range 0–16) mm Hg (*P* = .001) at the 6-month follow-up. Systolic blood pressure measured a median of 120 (range 96–151) mm Hg in patient group and 101 (range 72–123) mm Hg in controls (*P* < .001). During the 12-month follow-up, it decreased to a median of 105 (range 83–117) mm Hg in patients (*P* = .001 compared with baseline) and did not differ from that of controls.

At baseline, serum levels of ANPN were higher than in controls (Figure 1; Table 2). After relief of CoA, a significant reduction occurred in levels of peptides as compared with baseline. At the 6-month follow-up, levels of both ANPN and NT-proBNP were higher than in controls but after one year, the levels were similar to controls (Table 2).

**Figure 4:**
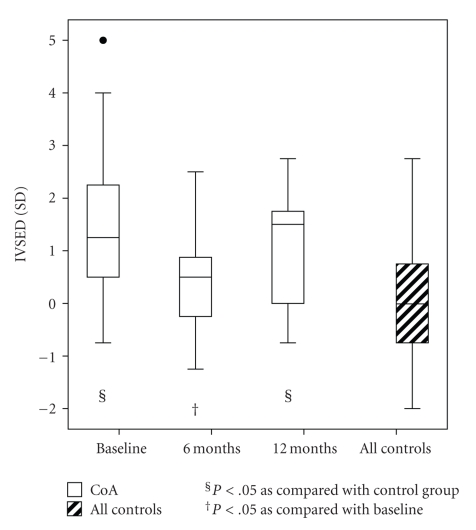
The *z*-score (SD) of end-diastolic thickness of interventricular septum (IVSED) in patients with coarctation of the aorta (CoA) and in controls. Whiskers at the top and bottom of each box indicate maximum and minimum; the top and bottom of each box the third and first quartiles; line through the box the median. Solid circle is an outlier.

Six months after treatment, the *z*-score of the end-diastolic thickness of the interventricular septum had decreased, but it increased again by the 12-month follow-up (Figure 4). The end-diastolic diameter of RV increased during follow-up (Table 3). In 3D echocardiography, the end-diastolic and systolic volumes of LV remained unchanged.

### 4.4. Comparison of Patient Groups at Baseline

Among patients older than 6 months, levels of NT-proBNP were the highest in children with PDA (*P* = .004 as compared with patients with CoA, *P* = .032 as compared with those with ASD), followed by those with ASD (*P* = ns as compared with patients with CoA), and finally those with CoA. ANPN levels at baseline were similar in all patient groups, but higher in these than in controls.

### 4.5. Correlations

At baseline, serum levels of ANPN and NT-proBNP correlated with 2D and 3D echocardiographic dimensions of RV and LV according to the loading condition. In the ASD group, serum levels of NT-proBNP correlated with the end-systolic volume of LV (*r* = 0.487, *P* = .025). In patients with PDA and volume overload of the left side of the heart, serum levels of ANPN and pro-BNP correlated with LV end-diastolic diameter (*r* = 0.513, *P* = .009, and *r* = 0.446, *P* = .025, resp.) and volume (*r* = 0.464, *P* = .020 and *r* = 0.409, *P* = .042, resp.) in 2D echocardiography. In patients with CoA, negative correlations were found between levels of ANPN and NT-proBNP and 3D echocardiographic end-diastolic volume of LV (*r* = −0.719, *P* = .003; and *r* = −0.744, *P* = .001, resp.).

## 5. Discussion

In this prospective follow-up study, we evaluated the influence of treatment for different types of loading conditions on serum levels of ANPN and NT-proBNP, and correlated peptide levels to 2D and 3D echocardiographic findings. ANPN levels were higher than in controls at baseline in all patient groups, and NT-proBNP in patients with ASD and PDA. Both peptide levels were elevated 6 months after treatment, and decreased thereafter. At the 6-month follow-up, the patients with PDA were the only group in whom the dimensions of the originally overloaded ventricle had normalized. The end-diastolic diameter of RV was larger in patients with ASD than in controls and the interventricular septum thicker in patients with CoA than in controls.

Shunting through the ASD causes underfilling of the LV, and volume overload of the RV disturbs ventricular septal motion [24, 25]. The diastolic and systolic function of the LV can thus be mechanically disturbed [24]. Acute improvement in preload and early diastolic function of the LV after ASD closure depends on the degree of the RV volume overload and is associated with normalization of the ventricular septal motion and ventricular interdependence [26]. LV symmetry increases months after surgical ASD repair [27, 28].

In some studies, end-diastolic diameter of the RV has decreased after ASD closure, but remained enlarged as compared with that of controls [15, 29–32]. Some studies have demonstrated that after closure of the defect, end-diastolic diameters and volumes of LV and RV change towards normal values [33, 34]. Similarly, in our patients, size of LV increased during follow-up, and no difference was present between patients and controls 6 months after treatment. The end-diastolic diameter of RV decreased, but remained larger than in controls up to 12 months after treatment [35].

In pediatric patients with ASD, blood levels of ANPN have been shown to correlate with the degree of shunting [36]. After closure of ASD, levels of ANPN and volume of RV have decreased [13]. Serum levels of ANPN have been higher than in matched controls up to 35 years after surgical closure of ASD [37]. On the other hand, plasma levels of ANP and BNP have normalized during a 3-month follow-up after device closure of ASD in children [15]. In our study, six months after ASD closure, the difference between patients and controls in serum levels of ANPN and NT-proBNP was still evident, but not after 12 months. Closure of ASD redirects blood flow to LV. Therefore, in our study, the size of LV increased. This may be why at 6 months the peptide levels had not started to decrease.

We have earlier shown that a day after PDA occlusion, serum levels of NT-proBNP increase, but decrease to control levels in 6 months [23]. Our finding of the highest levels of NT-proBNP in patients with PDA and volume overload of LV is in accordance with the previous studies [38, 39]. To our knowledge, no correlation between echo parameters and peptide levels has been published in patients with left-sided volume overload caused by PDA. In our patients with PDA, a significant correlation was found between serum levels of ANPN and NT-proBNP and the end-diastolic diameter and volume of LV.

Despite early and apparently successful repair of CoA, myocardial hypertrophy may persist, even in the presence of LV remodeling [40–43]. In our study, after treatment for CoA, the thickness of the interventricular septum after the first 6 months decreased and the diameter of RV increased [44]. At baseline, children with CoA had higher levels of ANPN than controls, but the difference in levels of NT-proBNP failed to reach statistical significance. The difference between patients and controls in levels of both peptides was evident at the 6-month follow-up. Serum levels of ANPN and NT-proBNP correlated negatively with size of LV in 3D echocardiography. This may reflect diastolic ventricular dysfunction and restrictive physiology of the ventricles even after repair, leading to dilatation of atria.

When different patient groups were compared at baseline, no differences emerged in levels of ANPN and NT-proBNP between volume and pressure overload of LV. Volume overload of LV caused higher levels of NT-proBNP than that of RV, but no differences in levels of ANPN were seen. Others have found higher plasma levels of ANP in volume overload of LV (VSD and PDA) than in right- sided volume overload [12]. In our patients with ASD and CoA, no difference was evident. The effects of volume overload on the levels of ANPN and BNP have been investigated in patients with VSD and PDA, but no follow-up studies are available. Plasma levels of BNP have been demonstrated to correlate with systolic RV pressure in children with volume overload of RV [45].

Plasma levels of BNP have been shown to correlate with right atrial and ventricular pressures in a child population consisting of different loading conditions and a wide age range [14]. In adults, plasma levels of BNP are higher in patients with acquired LV dysfunction. They are also higher in those with congenital heart defect and RV failure than in patients without structural heart disease [46]. ANP and BNP may have different regulation of gene expression depending on the form and localization of the stress exerted on the heart [47]. This may explain why in our study the correlation between serum levels of natriuretic peptides and echocardiographic dimensions varied under different loading conditions.

The most significant limitation to this study is that the number of patients in each group of patients is small and therefore we cannot compare the patients undergoing surgical and percutanoeus treatment. The age range is wide in patient groups and controls. The controls were examined only once. However, we assumed that the levels of peptides would not change significantly during one year. 

In conclusion, serum levels of both ANPN and NT-proBNP are higher in patients with ASD or PDA than in controls. In patients with CoA, ANPN levels are higher than in controls. After treatment, peptide levels decrease and some remodeling of the originally loaded ventricle takes place during within 6–12 months. Natriuretic peptide levels correlate with echocardiographic findings and reflect the hemodynamic loading condition. Therefore, natriuretic peptides might be useful in the pre-and postoperative follow-up of these patient groups.

## Figures and Tables

**Table 1:** Characteristics of subjects in the study groups at baseline. Values are expressed as median (range).

Group	*N*	Gender (m/f)	Age (years)	No. of patients aged less than 6 months	Weight (kg)	Height (cm)	BSA (m²)
Controls	76	32/44	5.4	12	20.4	113.8	0.79
			(0.0–15.6)		(3.1–66.5)	(48.0–174.6)	(0.19–1.81)

Patients	21	7/14	5.2	0	20.0	105.1	0.78
with ASD			(2.3–14.9)		(12.4–59.0)	(83.0–163.0)	(0.53–1.63)

Patients	25	11/14	2.6	0	13.0	87.5	0.54
with PDA			(1.0–10.6)		(6.9–32.8)	(70.0–139.5)	(0.36–1.14)

Patients	15	8/7	2.0	7	14.0	91.5	0.59
with CoA			(0.0–15.5)		(3.4–56.6)	(49.5–164.0)	(0.20–1.57)

ASD: atrial septal defect; BSA: body surface area; CoA: coarctation of the aorta;

PDA: patent ductus arteriosus.

**Table 2:** Serum levels of ANPN and NT-proBNP in patients measured at baseline and at 6 and 12 months' follow-up, and in controls. Values are expressed as median (range).

	*N*	ANPN (nmol/L)	NT-proBNP (ng/L)
		Median (range)	Median (range)

Controls	76	0.30 (0.10–3.70)	68 (14–3224)
Controls	64	0.28 (0.10–0.61)	57 (14–157)
>0.5 year			
ASD	21	0.44^§^ (0.15–1.40)	79^§^ (11–245)
baseline			
ASD	20	0.40^§^ (0.17–0.71)	96^§^ (17–202)
6 months			
ASD	21	0.31^†^ (0.10–0.70)	58 (17–260)
1 year			
PDA	25	0.37^§^ (0.21–1.21)	141^§^ (36–974)
baseline			
PDA	25	0.37^§†^ (0.14–0.58)	71^§†^ (21–228)
6 months			
CoA	15	0.60^§^ (0.16–16.30)	128 (5–35001)
baseline			
CoA	15	0.46^§†^ (0.09–1.20)	86^§†^ (9–441)
6 months			
CoA	13	0.34^†^ (0.09–0.82)	60^†^ (32–309)
12 months			

ANPN: N-terminal proatriopeptide; ASD: atrial septal defect; CoA: coarctation of the aorta;

Mos: months; NT-proBNP: N-terminal probrain natriuretic peptide; PDA: patent ductus arteriosus;

^§^ *P* < .05 as compared with control group > 0.5 years except CoA group at baseline is compared with whole control group;

^†^ *P* < .05 as compared with baseline.

**Table 3:** Two- and three-dimensional echocardiographic findings in control subjects and in groups of children with ASD, PDA, and CoA, measured at baseline and at 6 and 12 months after repair. Values are given as median (range).

Diagnosis	Controls	ASD			PDA		CoA		
		baseline	6 months	12 months	baseline	6 months	baseline	6 months	12 months
			f-u	f-u		f-u		f-u	f-u
*N*	76	21		21	25	25	15	15	13
IVSED	0.00	0.25	0.00	0.50^†^	0.50	0.25	1.25^§^	0.50^†^	1.50^§^
(SD)	(−2.00–2.75)	(−1.00–3.75)	(−2.00–1.25)	(−1.50–2.50)	(−1.75–4.50)	(−1.75–4.25)	(−0.75–5.00)	(−1.25–2.50)	(−0.00–2.75)
LVPWED	−0.50	−0.25	−0.25	0.00^§†^	−0.50	−0.25	0.25^§^	0.00	0.75^§^
(SD)	(−2.25–2.00)	(−1.50–2.00)	(−2.25–1.00)	(−1.25–1.25)	(−2.00–2.75)	(−1.25–2.25)	(−2.50–1.75)	(−1.50–1.50)	(−0.75–1.75)
RVEDD	0.25	4.75^§^	1.25^§†^	2.25^§†^	0.125	0.625^§†^	−0.375	0.75^†^	0.50
(SD)	(−1.75–2.50)	(2.25–9.75)	(−1.50–5.50)	(−0.25–3.50)	(−1.75–2.75)	(−1.75–3.50)	(−2.25–1.75)	(−2.00–2.00)	(−2.25–1.75)
LVEDD	−0.25	−1.50^§^	0.00^†^	−0.50^†^	0.75^§^	0.00^†^	0.75^§^	1.00^§^	1.00^§^
(SD)	(−1.75– 2.00)	(−3.75–0.00)	(−3.00–1.25)	(−1.75–1.25)	(−1.25–3.75)	(−2.00–1.75)	(−1.25–4.25)	(−1.00–3.25)	(−0.50–2.00)
LVEDV	62.1	47.2^§^	63.0^†^	60.4^†^	78.4^§^	64.2^†^	75.0^§^	65.8	72.5^§^
(mL/m²)	(36.9–87.7)	(27.6–67.1)	(35.5–83.1)	(43.5–92.9)	(44.7–117.1)	(37.6–89.5)	(47.9–115.6)	(49.8–98.5)	(52.5–82.4)
LVESV	19.3	13.2^§^	18.5^†^	19.4^†^	24.5^§^	19.5^†^	19.7	18.4	18.5
(mL/m²)	(7.7–30.6)	(7.4–33.2)	(8.0–28.2)	(12.5–31.8)	(12.3–45.0)	(11.9–33.3)	(12.8–68.1)	(8.9–37.4)	(9.2–32.8)
LVED3D	56.2	49.2^§^	63.5^§†^	65.1^§†^	57.5	57.7	64.7	61.4	71.4^§^
(mL/m²)	(32.0–89.5)	(31.0–65.3)	(36.5–93.8)	(40.0–107.6)	(43.6–99.4)	(41.0–84.2)	(35.6–145.3)	(30.7–131.1)	(35.7–121.2)
LVES3D	28.5	24.1^§^	31.0^†^	33.8^§†^	29.8	27.6	38.1	28.8	30.2
(mL/m²)	(14.1–52.8)	(16.2–38.8)	(17.5–45.7)	(18.1–48.9)	(19.4–54.1)	(18.1–37.6)	(16.8–93.5)	(9.3–80.1)	(14.9–56.5)

ASD: atrial septal defect, CoA: coarctation of the aorta; IVSED (SD): *z*-score (SD) of the end-diastolic thickness of interventricular septum; LVEDD (SD): *z*-score (SD) of the end-diastolic diameter of left ventricle; LVESD (SD): *z*-score (SD) of the end-systolic diameter of left ventricle; LVEDV: end-diastolic volume of left ventricle adjusted for body surface area; LVESV: end-systolic volume of left ventricle adjusted for body surface area; LVED3D: end-diastolic volume of left ventricle adjusted for body surface area measured by 3D echocardiography; LVES3D: end-systolic volume of left ventricle adjusted for body surface area measured by 3D echocardiography, LVPWED (SD): *z*-score (SD) of the end-diastolic thickness of left ventricular posterior wall; PDA: patent ductus arteriosus; RVEDD (SD): *z*-score (SD) of the end-diastolic diameter of right ventricle; SD: standard deviation of published normal values;

^§^ *P* < .05 as compared with control group;

^†^ *P* < .05 as compared with baseline.
